# Supplementary material for: Growth and rapid succession of methanotrophs effectively limit methane release during lake overturn
Source: Commun Biol. 2020 Mar 6;3:108. doi: 10.1038/s42003-020-0838-z (PMC7060174; doi:10.1038/s42003-020-0838-z)
Supplement: Supplementary file 1 — Supplementary Information [file 42003_2020_838_MOESM1_ESM.pdf]

Supplementary Material for

Growth and rapid succession of methanotrophs effectively limit methane release during lake overturn

Supplementary Figures

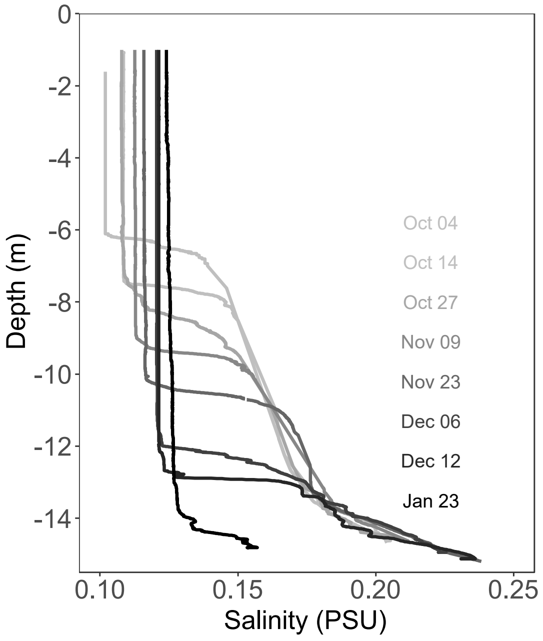

Supplementary Figure 1 Salinity profiles taken with the profiling in-situ analyser (PIA). Salinity profiles at each sampling date during the lake overturn which continued from October to December. The higher salinity in the bottom water allowed for an inverse stratification on Dec 12 (see Fig. 1b).

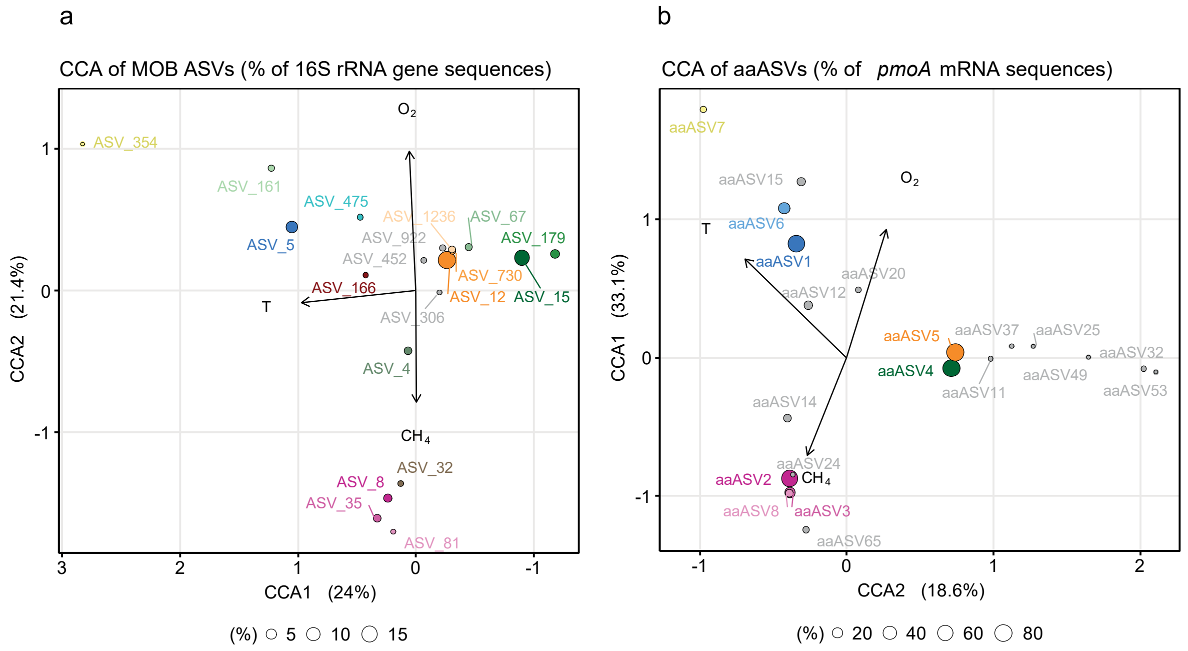

Supplementary Figure 2 Canonical correspondence analysis (CCA) calculated based on a Chi-square dissimilarity matrix using the proportion of 16S rRNA gene sequences identified as MOB and physico-chemical parameters (temperature (T), oxygen (O_2) and methane (CH_4). The CCA was calculated for **a** 16S rRNA gene MOB ASVs and **b** *pmoA* mRNA aaASVs of all samples during lake overturn. Percentage of explained variance is given next to each axis. Colors are ASV/aaASV specific and correspond to the colours used in Figures 2 and 3 of the main text. The dot size visualizes the maximum percentage of the ASV/aaASV during lake overturn.

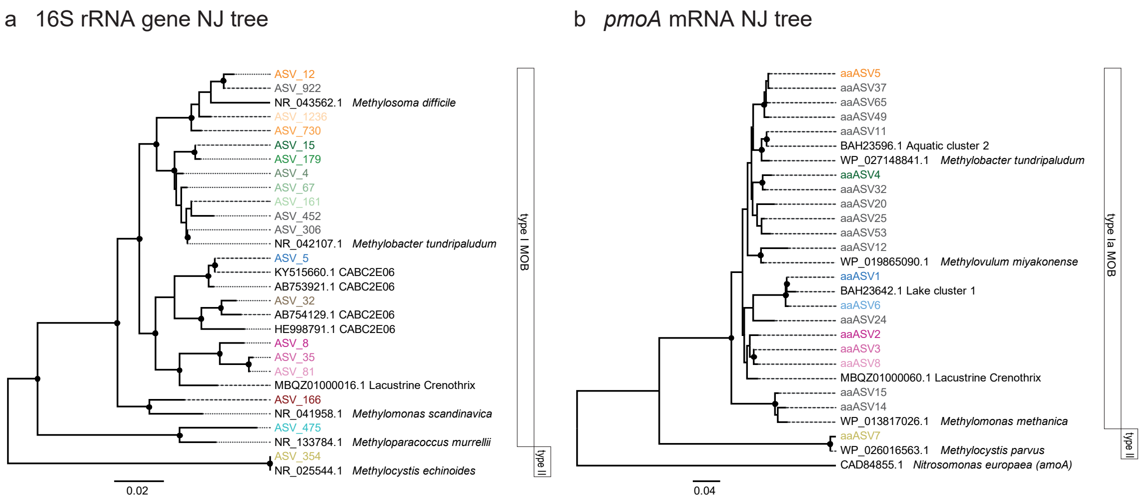

Supplementary Figure 3 Phylogenetic placement of MOB 16S rRNA genes and *pmoA* amino acid sequences found in this study with reference sequences from cultivated and uncultivated MOB. Neighbor joining (NJ) trees were inferred with MEGA7 based on **a** partial 16S rRNA gene sequences (423bp) and Jukes-Cantor evolutionary distance, and **b** partial *pmoA* amino acid sequences (155 positions) and Poisson correction method. Black dots indicate nodes with a bootstrap value above 0.7 (10 000 bootstrap replicates). Colors are ASV/aaASV specific and correspond to the colours used in Figures 2 and 3 of the main text. Reference sequences are given with accession numbers to the left of the species name, or cluster name in case of uncultivated MOB. Scale bars represent changes per nucleotide or amino acid position.

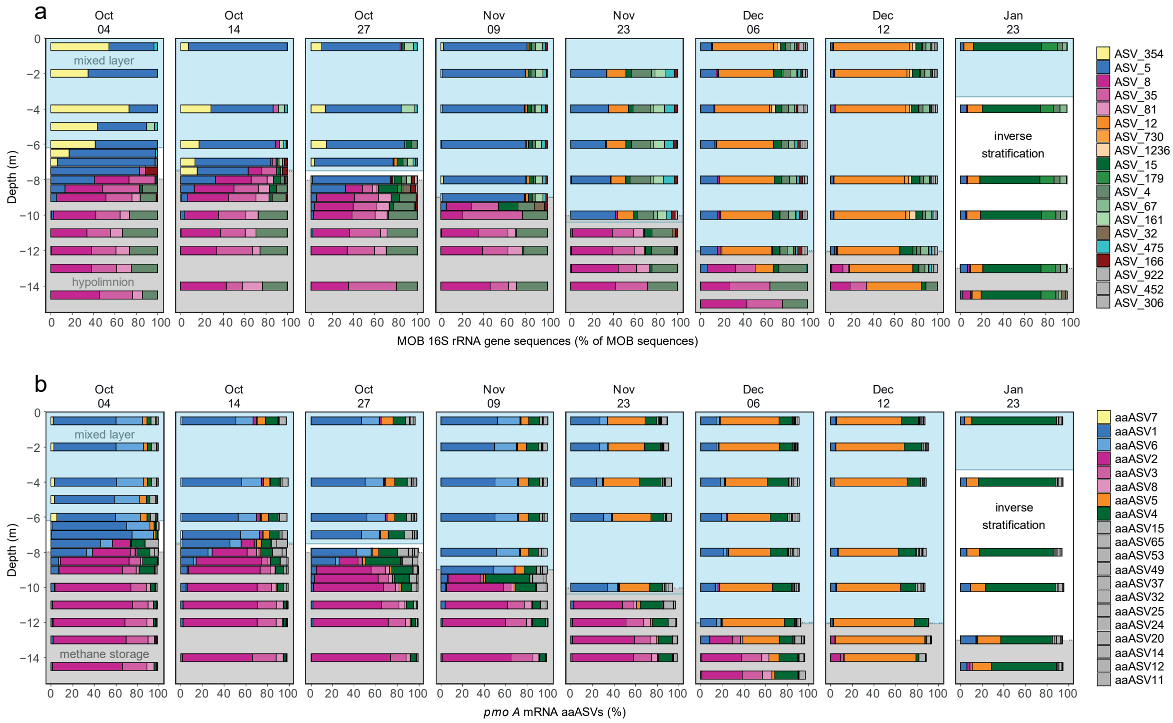

Supplementary Figure 4 Depth distribution and dynamics within the MOB assemblage during lake overturn. The mixed layer depth increased with time (blue background), while the methane-rich bottom water (grey background) got gradually incorporated into the mixed layer. **a** proportion of MOB ASVs among all identified MOB based on 16S rRNA gene amplicon sequencing **b** Proportion of *pmoA* mRNA aaASVs based on amplicon sequencing. Colors are ASV/aaASV specific and correspond to the colours used in Figures 2 and 3 of the main text. ASVs and aaASVs which, based on multiple lines of evidence (see methods), are thought to originate from the same organism are encoded with the same color.

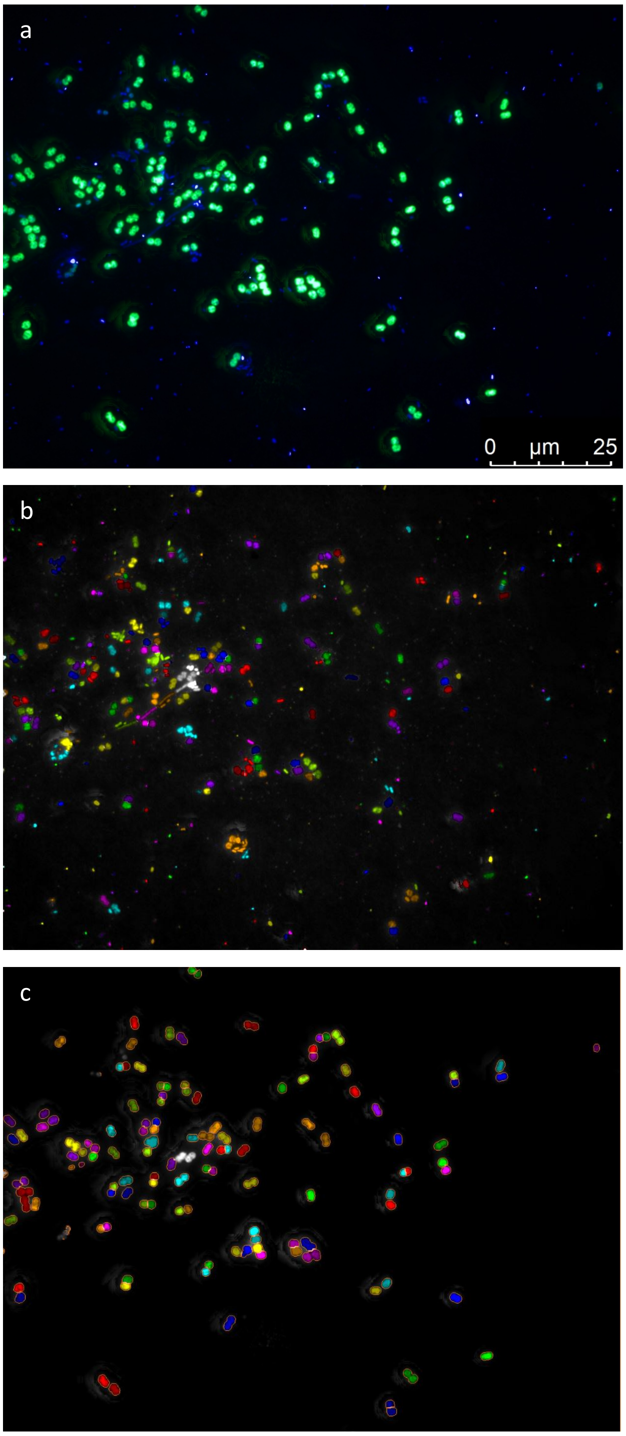

Supplementary Figure 5 Exemplary cell identification of DAPI-stained cells and CARD-FISH-stained cells using daime**.** **a-c** show an identical field of view. **a** shows DAPI-stained cells (blue) and CARD-FISH-stained cells (green) labelled with probes Mg84, Mg705 and Mg669 in the original micrograph. **b** and **c**, respectively, show the DAPI-stained cells and CARD-FISH-stained cells identified with daime. The colours in **b** and **c** indicate the identified cells and are selected at random. To identify the cells, original images were cropped to a uniform size of 1300 x 990 px (120.90 µm x 92.07 µm). Image histograms were manually stretched to reduce background noise and increase signal contrast. To identify individual objects (cells), images were segmented with an edge detection algorithm (Daims et al., 2006), chosen to avoid an illumination thresholding bias caused by the pronounced differences in signal brightness. Objects with a diameter smaller than the filter pore size (0.2 µm) were excluded. Touching cells were split with a watershed segmentation-based algorithm (Daims et al., 2006). Artefacts (hybridised cell signals with no corresponding DAPI signal) were excluded, applying a minimum congruency threshold of 1 %. If necessary, remaining artefacts, falsely segmented cells, large cell aggregates and image pairs with high background noise were manually excluded, retaining a minimum of 20 image pairs per filter piece for further analysis. Falsely unidentified or excluded hybridised cell signals caused by DAPI fluorochrome quenching, were manually included. The same procedure was applied to all images to ensure comparability.

Supplementary Tables

Supplementary Table 1 Spearman rank correlation table of the MOB parameters and potential methane oxidation rate showing the strength of correlation. p-values were <0.001 after adjusting for multiple comparisons in R using the Benjamini & Hochberg (BH) method (Benjamini, Y., and Hochberg, Y., 1995. Controlling the false discovery rate: a practical and powerful approach to multiple testing. *Journal of the Royal Statistical Society Series B*, 57, 289–300).

Spearman's rho	***pmoA*** **mRNA (copies ml^{-1})**	***pmoA*** **DNA (copies ml^{-1})**	**MOB 16S rRNA gene (%)**	**MOB 16S rRNA (%)**	**MOB cells (ml^{-1})**
**CH_4 oxidation rate (µM d^{-1})**	0.82 (n=83)	0.64 (n=83)	0.88 (n=83)	0.84 (n=83)	0.75 (n=69)
pmoA **mRNA (copies ml^{-1})**		0.83 (n=83)	0.90 (n=83)	0.81 (n=84)	0.83 (n=68)
pmoA **DNA (copies ml^{-1})**			0.75 (n=83)	0.62 (n=83)	0.79 (n=68)
**MOB 16S rRNA gene (%)**				0.91 (n=83)	0.82 (n=68)
**MOB 16S rRNA (%)**					0.69 (n=68)

Supplementary Table 2 Summary of measured mixed layer values used to produce Figure 3B and 3C. Abbreviations: Temp=Temperature, FCM = cells based on flow cytometry, pot. = potential, NA = missing values. Measurements smaller than the limit of quantification (LOQ) are indicated with "<" and the value of LOQ. In case of methane the lowest calibration concentration is shown. LOQ of nitrate was derived from the FIA instrument baseline.

Date DD.MM.YYYY	Layer	Depth (m)	Temp (°C)	Oxygen ($\mu mol\ l^{-1}$)	Methane ($\mu mol\ l^{-1}$)	Nitrate ($\mu mol\ l^{-1}$)	FCM (cells ml^{-1})	Pot. methane oxidation rate ($\mu mol\ l^{-1}\ d^{-1}$)
04.10.2016	mixed	0.50	18.4	413	0.3	< 4	6.3E+06	NA
04.10.2016	mixed	2.00	18.5	412	0.3	< 4	5.8E+06	0.2
04.10.2016	mixed	4.00	18.5	412	0.3	< 4	5.4E+06	0.1
04.10.2016	mixed	5.00	18.4	412	0.2	< 4	5.7E+06	0.2
04.10.2016	mixed	6.00	18.4	412	0.3	< 4	5.9E+06	0.2
	**MEDIAN:**	**n=5**	**18.4**	**412**	**0.3**	**< 4**	**5.8E+06**	**0.2**
14.10.2016	mixed	0.50	14.4	279	0.6	< 4	6.2E+06	0.5
14.10.2016	mixed	4.00	14.4	276	0.6	< 4	6.2E+06	0.6
14.10.2016	mixed	6.00	14.4	277	<0.05	< 4	6.1E+06	0.4
14.10.2016	mixed	7.00	14.4	273	0.7	< 4	6.0E+06	0.5
	**MEDIAN:**	**n=4**	**14.4**	**277**	**0.6**	**< 4**	**6.1E+06**	**0.5**
27.10.2016	mixed	0.50	13.1	290	0.1	< 4	5.2E+06	0.3
27.10.2016	mixed	4.00	13.0	287	0.1	< 4	5.8E+06	0.7
27.10.2016	mixed	6.00	13.0	285	0.2	< 4	5.2E+06	0.5
27.10.2016	mixed	7.00	13.0	280	0.2	< 4	5.3E+06	1.0
	**MEDIAN:**	**n=4**	**13.0**	**286**	**0.1**	**< 4**	**5.3E+06**	**0.6**
09.11.2016	mixed	0.50	10.7	272	1.4	4.6	5.6E+06	2.5
09.11.2016	mixed	2.00	10.7	271	1.3	5.2	5.4E+06	2.5
09.11.2016	mixed	4.00	10.7	270	1.0	5.4	5.5E+06	2.4
09.11.2016	mixed	6.00	10.7	269	1.0	5.6	5.4E+06	3.0
09.11.2016	mixed	8.00	10.7	270	1.1	6.1	5.2E+06	2.6
	**MEDIAN:**	**n=5**	**10.7**	**270**	**1.1**	**5.4**	**5.4E+06**	**2.5**
23.11.2016	mixed	0.50	8.9	248	0.1	8.0	NA	2.2
23.11.2016	mixed	2.00	8.9	245	0.1	8.5	NA	2.5
23.11.2016	mixed	4.00	8.9	244	0.1	8.4	NA	2.8
23.11.2016	mixed	6.00	8.9	245	<0.05	8.3	NA	2.4
23.11.2016	mixed	8.00	8.9	243	<0.05	8.4	NA	2.7
	**MEDIAN:**	**n=5**	**8.9**	**245**	**0.1**	**8.4**	**NA**	**2.5**
06.12.2016	mixed	0.50	7.0	181	0.4	8.2	2.5E+06	5.3
06.12.2016	mixed	2.00	7.0	179	0.7	8.5	2.5E+06	5.3
06.12.2016	mixed	4.00	7.0	179	0.7	8.7	2.5E+06	5.6
06.12.2016	mixed	6.00	7.0	180	0.5	9.1	2.5E+06	5.4
06.12.2016	mixed	8.00	7.0	178	0.9	8.7	2.5E+06	5.8
06.12.2016	mixed	10.00	7.0	177	0.5	8.7	2.5E+06	5.2
	**MEDIAN:**	**n=6**	**7.0**	**179**	**0.6**	**8.7**	**2.5E+06**	**5.3**
12.12.2016	mixed	0.50	6.1	175	<0.05	8.8	2.1E+06	7.2
12.12.2016	mixed	2.00	6.1	175	<0.05	8.8	2.1E+06	6.4
12.12.2016	mixed	4.00	6.1	173	<0.05	9.3	1.9E+06	6.2
12.12.2016	mixed	8.00	6.1	175	0.3	9.1	2.1E+06	7.8
12.12.2016	mixed	10.00	6.0	172	<0.05	9.0	1.9E+06	6.3
	**MEDIAN:**	**n=5**	**6.1**	**175**	**<0.05**	**9.0**	**2.1E+06**	**6.4**
**23.01.2017**	**mixed**	**0.50**	**1.4**	**329**	**0.1**	**10.6**	**5.0E+06**	**2.0**
	**MEDIAN:**	**n=1**						
