# Supplementary material for: Growth and rapid succession of methanotrophs effectively limit methane release during lake overturn
Source: Commun Biol. 2020 Mar 6;3:108. doi: 10.1038/s42003-020-0838-z (PMC7060174; doi:10.1038/s42003-020-0838-z)
Supplement: Supplementary file 2 — Reporting Summary [file 42003_2020_838_MOESM2_ESM.pdf]

nature research

Corresponding author(s): Helmut Bürgmann

Last updated by author(s): Nov 13, 2019

Reporting Summary

Nature Research wishes to improve the reproducibility of the work that we publish. This form provides structure for consistency and transparency in reporting. For further information on Nature Research policies, see Authors & Referees and the Editorial Policy Checklist.

Statistics

For all statistical analyses, confirm that the following items are present in the figure legend, table legend, main text, or Methods section.

n/a	Confirmed	
☐	☒	The exact sample size (*n*) for each experimental group/condition, given as a discrete number and unit of measurement
☐	☒	A statement on whether measurements were taken from distinct samples or whether the same sample was measured repeatedly
☐	☒	The statistical test(s) used AND whether they are one- or two-sided<br>*Only common tests should be described solely by name; describe more complex techniques in the Methods section.*
☐	☒	A description of all covariates tested
☐	☒	A description of any assumptions or corrections, such as tests of normality and adjustment for multiple comparisons
☐	☒	A full description of the statistical parameters including central tendency (e.g. means) or other basic estimates (e.g. regression coefficient) AND variation (e.g. standard deviation) or associated estimates of uncertainty (e.g. confidence intervals)
☒	☐	For null hypothesis testing, the test statistic (e.g. *F*, *t*, *r*) with confidence intervals, effect sizes, degrees of freedom and *P* value noted<br>*Give P values as exact values whenever suitable.*
☒	☐	For Bayesian analysis, information on the choice of priors and Markov chain Monte Carlo settings
☒	☐	For hierarchical and complex designs, identification of the appropriate level for tests and full reporting of outcomes
☒	☐	Estimates of effect sizes (e.g. Cohen's *d*, Pearson's *r*), indicating how they were calculated

Our web collection on statistics for biologists contains articles on many of the points above.

Software and code

Policy information about availability of computer code

Data collection	No specialized software was used for data collection.
Data analysis	Free software R (v3.4.1) was used to analyze the sequencing data (available at the European Nucleotide Archive). Amplicon sequence variants were inferred with DADA2 (v1.6.0). Further analyses and plots were done in R (3.5.0) with phyloseq (v1.24.2) and vegan (v2.5.2.). Further ggplot2 (v3.2.0) was used for data visualization. Daime (v2.0) was used for processing CARD-FISH images.

For manuscripts utilizing custom algorithms or software that are central to the research but not yet described in published literature, software must be made available to editors/reviewers. We strongly encourage code deposition in a community repository (e.g. GitHub). See the Nature Research guidelines for submitting code & software for further information.

Data

Policy information about availability of data

All manuscripts must include a data availability statement. This statement should provide the following information, where applicable:

- Accession codes, unique identifiers, or web links for publicly available datasets
- A list of figures that have associated raw data
- A description of any restrictions on data availability

The sequencing files have been submitted to the European nucleotide archive under the project number PRJEB32413 . All other data is available from the Eawag Research Data Institutional Collection (https://data.eawag.ch/dataset/growth-succession-of-methanotrophs-limit-methane-release-lake-overturn).

Field-specific reporting

Please select the one below that is the best fit for your research. If you are not sure, read the appropriate sections before making your selection.

☐ Life sciences ☐ Behavioural & social sciences ☒ Ecological, evolutionary & environmental sciences

For a reference copy of the document with all sections, see nature.com/documents/nr-reporting-summary-flat.pdf

Ecological, evolutionary & environmental sciences study design

All studies must disclose on these points even when the disclosure is negative.

Study description	We sampled the lake overturn period in Lake Rotsee (Switzerland) at eight time points. We used 16S rRNA gene amplicon sequencing, pmoA mRNA sequencing and qPCR, CARD-FISH and potential methane oxidation rate measurements to investigate succession, growth and methane oxidation capacity of the MOB assemblage during lake overturn.
Research sample	We used freshwater lake water column samples taken with the profiling in-situ analyzer at different depths of Lake Rotsee, which were stored processed and analyzed as described in the methods.
Sampling strategy	We did not predetermine sample size. But we installed a thermistor string giving temperature measurements every few hours. This was done in order not to miss any major mixing event of the lake and to schedule the sampling dates.
Data collection	Environmental data were collected partially on-site with sensors of the profiling in-situ analyzer (depth, temperature, salinity, oxygen concentration). Further variables were measured in the laboratory (methane concentration, nitrate concentration, cell numbers). Incubations to measure potential methane oxidation rates were set-up in the laboratory. CARD-FISH, amplicon sequencing and qPCR (DNA+RNA) were performed to analyze the response of methane-oxidizing bacteria to lake overturn
Timing and spatial scale	Sampling was done on eight dates from October 4th, 2016 to January 23rd, 2017. The sampling period was chosen to begin at the end of the summer stratification period (as the starting situation) with maximal methane accumulation in the hypolimnion and sampling continued until the end of the subsequent lake overturn.
Data exclusions	DNA removal from the RNA sample was checked by gel electrophoresis after PCR (16S rRNA gene, 27f/1492r, 35 cycles) and if required, a second digestion was performed. If the second digestion was not sufficient to remove the DNA the sample was excluded (one sample). To increase readability of Fig. 2 two samples (Oct 04) are not shown (7.25 m and 6.75 m), which does not change data interpretation. Samples with less than 5000 reads were excluded from the amplicon analysis. (see methods section)
Reproducibility	The analysis details as well as the data are available in different repositories, to facilitate transparency and reproducibility. Because this is an observational environmental study experimental replication was not possible. As this is an observational study, repeated experimental attempts are not applicable.
Randomization	Because we attempted to cover the lake overturn with our sampling regime randomization was not applicable.
Blinding	Not relevant for this study.

Did the study involve field work? ☒ Yes ☐ No

Field work, collection and transport

Field conditions	Lake Rotsee was sampled on 8 day during mostly calm and dry conditions from Oct 4, 2016 to Jan 23, 2017. Further information on field conditions (water temperatures) are given in Supplementary table 2.
Location	Lake Rotsee (47.072 N and 8.319 E) in Central Switzerland, elevation: 419m, maximum water depth: 16 m
Access and import/export	Rotsee is a small, easily accessible lake, situated relatively close to our institute (Eawag) in Switzerland and frequently sampled by Eawag. Permission for access and to take samples from Lake Rotsee and to install reserach equipment was obtained from the local authorities (City of Lucerne). Rotsee was accessed with a research boat owned by Eawag.
Disturbance	No disturbance was caused by taking samples from the lake.

Reporting for specific materials, systems and methods

We require information from authors about some types of materials, experimental systems and methods used in many studies. Here, indicate whether each material, system or method listed is relevant to your study. If you are not sure if a list item applies to your research, read the appropriate section before selecting a response.

Materials & experimental systems

n/a	Involved in the study
☒	☐ Antibodies
☒	☐ Eukaryotic cell lines
☒	☐ Palaeontology
☒	☐ Animals and other organisms
☒	☐ Human research participants
☒	☐ Clinical data

Methods

n/a	Involved in the study
☒	☐ ChIP-seq
☐	☒ Flow cytometry
☒	☐ MRI-based neuroimaging

Flow Cytometry

Plots

Confirm that:

☐ The axis labels state the marker and fluorochrome used (e.g. CD4-FITC).

☐ The axis scales are clearly visible. Include numbers along axes only for bottom left plot of group (a 'group' is an analysis of identical markers).

☐ All plots are contour plots with outliers or pseudocolor plots.

☐ A numerical value for number of cells or percentage (with statistics) is provided.

Methodology

Sample preparation	Water samples for flow cytometry to determine total microbial cell counts were fixed with formaldehyde and stained with SYBR Green I (Thermo Fisher Scientific, Waltham, MA, USA) at 37°C for 15 min. Particles were counted on an Accuri C6 flow cytometer (BD Biosciences, San Jose, CA, USA).
Instrument	Accuri C6 flow cytometer (BD Biosciences, San Jose, CA, USA)
Software	BD Accuri C6 Software
Cell population abundance	Flow cytometry was used to provide total microbial cell counts according to an established method. No sophisticated delineations or groupings were done.
Gating strategy	The same gate was used for all samples. The gating follows well-established published protocols (Reference given in manuscript, Proctor et al. 2018)

☐ Tick this box to confirm that a figure exemplifying the gating strategy is provided in the Supplementary Information.
